# Circulating MicroRNAs in Idiopathic Pulmonary Fibrosis: A Narrative Review

**DOI:** 10.3390/cimb46120821

**Published:** 2024-12-04

**Authors:** Marisa Denisse Colin Waldo, Xochipilzihuitl Quintero-Millán, Maria Cristina Negrete-García, Víctor Ruiz, Bettina Sommer, Dámaris P. Romero-Rodríguez, Eduardo Montes-Martínez

**Affiliations:** 1 Molecular Biology Laboratory, Department of Research in Pulmonary Fibrosis, National Institute of Respiratory Diseases “Ismael Cosío Villegas”, Calzada de Tlalpan 4502, Col. Sección XVI, Mexico City 14080, Mexico; 2 Bronchial Hyperreactivity Research Department, National Institute of Respiratory Diseases “Ismael Cosío Villegas”, Calzada de Tlalpan 4502, Col. Sección XVI, Mexico City 14080, Mexico; 3 Conahcyt National Laboratory for Research and Diagnosis by Immunocytofluorometry (LANCIDI), National Institute of Respiratory Diseases “Ismael Cosío Villegas”, Calzada de Tlalpan 4502, Col. Sección XVI, Mexico City 14080, Mexico

**Keywords:** microRNAs, biomarkers, idiopathic pulmonary fibrosis

## Abstract

Idiopathic pulmonary fibrosis (IPF) is a chronic, deathly disease with no recognized effective cure as yet. Furthermore, its diagnosis and differentiation from other diffuse interstitial diseases remain a challenge. Circulating miRNAs have been measured in IPF and have proven to be an adequate option as biomarkers for this disease. These miRNAs, released into the circulation outside the cell through exosomes and proteins, play a crucial role in the pathogenic pathways and mechanisms involved in IPF development. This review focuses on the serum/plasma miRNAs reported in IPF that have been validated by real-time PCR and the published evidence regarding the fibrotic process. First, we describe the mechanisms by which miRNAs travel through the circulation (contained in exosomes and bound to proteins), as well as the mechanism by which miRNAs perform their function within the cell. Subsequently, we summarize the evidence concerning miRNAs reported in serum/plasma, where we find contradictory functions in some miRNAs (dual functions in IPF) when comparing the findings in vitro vs. in vivo. The most relevant finding, for instance, the levels of miRNAs let-7d and miR-21 reported in the serum/plasma in IPF, correspond to those found in studies in lung fibroblasts and the murine bleomycin model, reinforcing the usefulness of these miRNAs as future biomarkers in IPF.

## 1. Introduction

Interstitial lung diseases are a complex group of diseases that can be divided into those of known etiology and those with unknown causes. Currently, ILDs can be classified as follows: idiopathic interstitial pneumonia (IIP), autoimmune ILDs, exposure-related ILDs (hypersensitivity pneumonitis, silicosis, asbestosis, etc.), ILDs with cyst and/or airspace filling and sarcoidosis. ILDs frequently develop progressive fibrosis (PF-ILDs) which implies a decrease in the clinical, physical and radiographic conditions of the patients, which is reflected in the deterioration of their quality of life and survival. Examples of PF-ILDs where the development of progressive fibrosis has been observed are hypersensitivity pneumonitis, rheumatoid arthritis, systemic sclerosis, idiopathic nonspecific interstitial pneumonia (iNISP) and acute fibrinous and organizing pneumonia (AFOP), among others. Interestingly, it has recently been suggested that some PF-ILDs may have clinical behavior similar to IPF, including the tomographic pattern of usual interstitial pneumonia (UIP), such as hypersensitivity pneumonitis and rheumatoid arthritis. IPF is the most common fibrosing disease of the lung and is the archetypal example of progressive pulmonary fibrosis characterized by the tomographic pattern of usual interstitial pneumonia (UIP). IPF is characterized by the excessive accumulation of extracellular matrix proteins that culminates in the destruction of the lung architecture. Due to IPF being a chronic, progressive, irreversible condition with limited treatment options (lethal within a few years), attempts have been made over the years to find a biomarker for this disease. For this reason, the objective of this narrative review is the analysis of the literature on the miRNAs reported in the serum/plasma of patients with IPF, selecting those miRNAs validated by individual PCR reactions and a subsequent review of the evidence reported in relation to the FPI [1,2,3,4,5,6,7].

## 2. Materials and Methods

**Figure 1:**
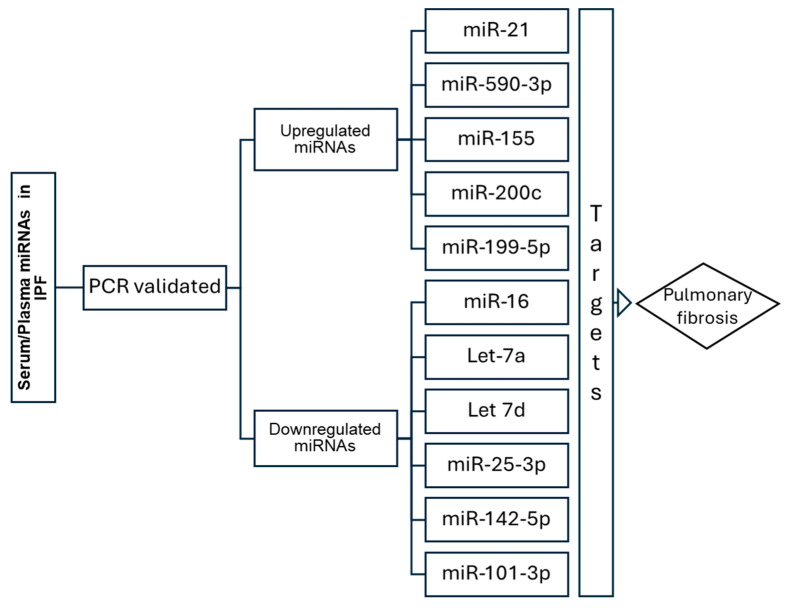
The miRNAs reported in the serum/plasma of patients with IPF are summarized in the figure above and include miRNAs with real-time PCR validation.

PubMed was the main information source for the development of the present work. The first inclusion criterion was to search for articles where miRNAs in the serum/plasma were evaluated in patients with IPF, using the following keywords: serum/plasma miRNAs idiopathic pulmonary fibrosis. From all the articles found, those validating miRNAs by independent reactions using real-time PCR were selected for further study. Afterwards, the miRNAs’ information was expanded by searching for experimental articles in which the target messenger RNAs (mRNAs) related to IPF, pulmonary fibrosis, and fibrosis were reported. In addition, the reports on the miRNAs and their target mRNAs were not only from AGO-CLIP-seq-type protocols (HITS-CLIP, PAR-CLIP, etc.) but also included experimental methods such as electrophoretic mobility shift assay, GFP reporter assay, luciferase reporter assay, and Western blot, among others (Figure 1 and Table 1). The figures were created using IbisPaintX Inc. version 12.2.8, BioRender.com, and Cytoscape software version 3.10.2. 

## 3. Idiopathic Pulmonary Fibrosis

**Figure 2:**
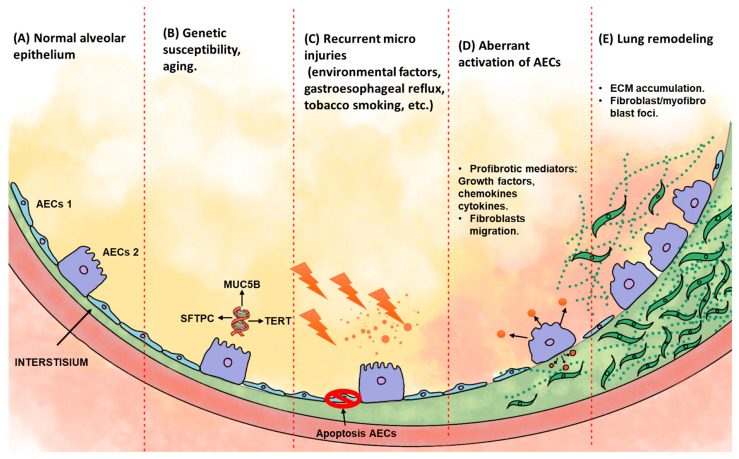
Figure illustrating IPF’s developmental stages. (**A**) Normal alveolar epithelium composed of alveolar epithelial cells type 1 (AECs 1) and alveolar epithelial cells type 2 (AECs 2). (**B**) Lung with genetic susceptibility and aging-related changes. (**C**) Recurrent micro injuries over time promote AEC apoptosis and (**D**) aberrant activation of AECs 2, producing profibrotic mediators. (**E**) These profibrotic molecules induce fibroblast migration and proliferation and myofibroblast differentiation, which produce exaggerated amounts of ECM proteins that contribute to lung remodeling.

IPF is a chronic, progressive, and lethal disease with an average survival of 3–5 years after diagnosis. The causes and mechanisms of IPF development remain unknown. It has been proposed that it is related to an epithelial-dependent process that starts with micro-injuries in alveolar epithelial cells (AECs), with subsequent aberrant activation of these cells. The disruption of the alveolar epithelial barrier causes hyperplasia of type II pneumocytes (cuboidalization) and AEC apoptosis. Damaged AECs produces many kinds of profibrotic mediators, such as transforming growth factor beta 1 (TGF-β1), connective tissue growth factor (CTGF), platelet-derived growth factor (PDFG), etc., that promote a profibrotic microenvironment in the lungs of IPF patients. This circumstance promotes migration and proliferation of lung fibroblasts, besides their differentiation into myofibroblast (activation). These cells produce excessive quantities of extracellular matrix (ECM) proteins like type I and III collagens, resulting in the destruction of the pulmonary architecture (Figure 2) [6,15,16,17,18,19].

The risk factors associated with IPF development include smoking, gastroesophageal reflux, chronic viral infections such as Epstein Barr and hepatitis C, a family history of interstitial lung diseases (rare variants in some genes such as MUC5B, TERT, SFTPC, etc.), and aging. The symptoms begin insidiously with chronic exertional dyspnea, cough, inspiratory bibasilar crackles, and digital clubbing without symptoms that suggest a multisystemic disease [4,20,21].

The natural history of IPF is heterogeneous; it can present chronic stable symptoms up to an acute respiratory exacerbation, with worsening dyspnea from days to weeks associated with a poor prognosis, causing 40% of the deaths in patients with IPF [22,23].

Additionally, some comorbidities may be present in patients with IPF, such as gastroesophageal reflux, chronic obstructive pulmonary disease (COPD), emphysema, obstructive sleep apnea, pulmonary hypertension (PH), cardiovascular (arrhythmia, atrial fibrillation, cardiac failure, ischemic heart disease, systemic arterial hypertension). In the case of PH, this is related to changes in the structure and function of the right ventricle, which would lead to heart failure. In this sense, some authors have suggested using techniques such as 2D global longitudinal strain (GLS) and two-dimensional speckle-tracking echocardiography as tools to evaluate patients with IPF, allowing for a better prognosis [24,25,26,27].

Another variation of IPF is rapidly progressive, and patients present a decrease in the forced vital capacity (FVC) and diffusion capacity of the lung for carbon monoxide (DLCO) of ≥10% and ≥15%, respectively, within the first 12 months after diagnosis, and this prognosis is severe. The diagnosis of this disease and its differentiation from other ILDs is challenging, and therefore, it is essential to accurately and promptly recognize it since inadequate treatment most probably increases morbidity and mortality [20,28].

## 4. Serum/Plasma Biomarkers in IPF

Several proteins have been measured in the serum of IPF patients to discover biomarkers that might simplify diagnosis and improve prognosis. The quest to find an IPF biomarker to differentiate this illness from other interstitial lung diseases like hypersensitivity pneumonitis, sarcoidosis, etc., remains a scientific research hotspot. In this context, some proteins evaluated as possible biomarkers of IPF include surfactant proteins A and D (SP-A and SP-D), Krebs von den Lungen 6 (KL6), C-C motif chemokines (CCL16, CCL18), matrix metalloproteinases (MMP7, and MMP28), renin and soluble (pro) renin receptor [29,30,31,32]. As of today, only two molecules have been corroborated to present different serum concentrations between IPF and other ILDs, renin and MMP7. Other molecules that have been measured in the serum of patients are micro RNAs, opening up promising new ways to identify IPF biomarkers.

## 5. miRNA Biogenesis and Mechanism of Action

**Figure 3:**
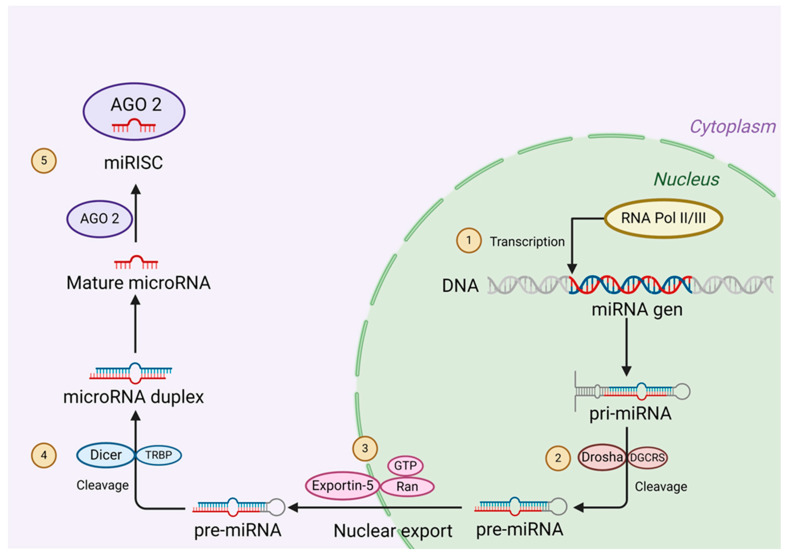
Figure depicting the miRNA biogenesis pathway. (1) The miRNA gene is transcribed through RNA polymerase II/III; this transcript is named pri-miRNA. (2) The pri-miRNA is matured by the protein complex formed by Drosha/DGCR8, producing a pre-miRNA. (3) The pre-miRNA is extruded from the nucleus via the nuclear pore complex exportin-5 to the cytoplasm. (4) The Dicer/TRBP complex processes the pre-miRNA. (5) The mature miRNA is directed to its target mRNA through the RNA-induced silencing complex (miRISC), formed by the miRNA and the AGO2 protein.

miRNAs are small RNAs with a length of 12–18 nucleotides (mature miRNA); they are capable of regulating many messenger RNAs (mRNAs) post-transcriptionally in two ways: by inhibiting mRNA translation or by inducing its disposal. miRNAs are non-coding RNAs found in the genome [33]. They are transcribed by RNA polymerase II (RNA pol II), generating an immature miRNA termed pri-miRNA, which needs to be processed until it becomes a mature miRNA [34]. The mechanism described as producing a mature miRNA begins when miRNA is transcribed by RNA pol II. This pri-miRNA is processed by the RNAse type III Drosha and DGCR8 (DiGeorge syndrome critical region 8) complex, producing a pre-miRNA of 70 nucleotides [35,36,37]. This pre-miRNA is exported outside the nucleus through the nuclear pore complex RAN GTP/exportin 5 [38,39]. Once in the cytoplasm, the complex formed by Dicer/protein kinase R/PACT processes the pre-miRNA, generating the mature miRNA (Figure 3). Subsequently, the mature miRNA is directed to its mRNA target through the miRISC protein complex, which consists of transactivating response RNA-binding protein (TRBP), that, in turn, recruits Argonaute 2 (AGO2), followed by the coupling of GW182 [40,41,42,43,44]. The miRISC, through AGO2, selects one of the two strands that are directed to the mRNA’s target [45].

miRISC leads the miRNA to the 3’untranslated region (3’UTR) of the mRNA, where the seed region is joined through Watson and Crick complementarity. This process induces two results: depending on the kind of mRNA, the translation of the mRNA will be inhibited either by preventing the formation of the pre-initiation of translation complex (eIF4G, ribosomal subunit formation) or by decoupling the translation machinery once the translation process has begun [41], or else the interaction of the miRNA with its target mRNA will produce the degradation of the mRNA [40,46].

## 6. Circulation of Exosomes and Circulating miRNAs

Exosomes can host different biomolecules, including nucleic acids (mRNA and miRNA) and various proteins, like enzymes, receptors, and transcription factors [47,48,49,50,51].

The biogenesis of exosomes begins through the biomolecules’ endocytosis into the cell by invagination of the plasma membrane, a cellular structure called “early-stage endosome” (ESE) [52]. ESE can follow two routes; it can form “recycling endosomes” or subsequently undergo a maturation process to become “late-classification endosomes” (LSE) [53,54], which finally leads to the formation of multivesicular bodies (MVBs). Its formation consists of a second intussusception within the endosomal membrane, thus forming the intraluminal vesicles (ILVs) [55].

**Figure 4:**
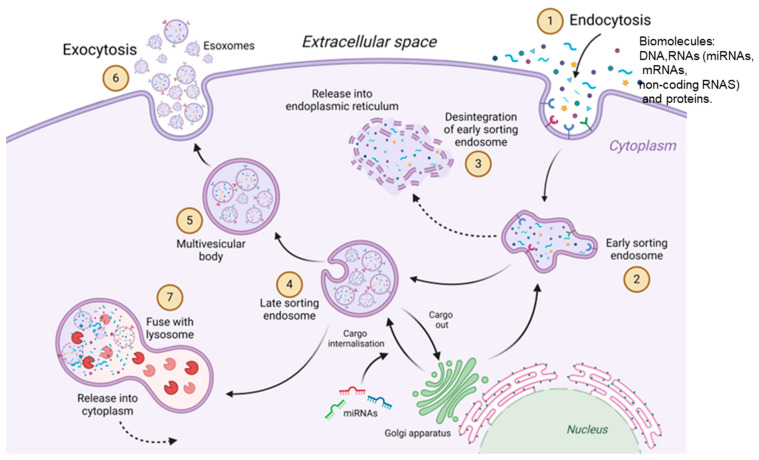
(1) The biogenesis of exosomes begins with the endocytosis of biomolecules such as nucleic acids: DNA, RNA (miRNAs, mRNAs, other non-coding RNAs) and proteins. (2) forming the early-stage endosome (ESE). (3, 4) Formation of late endosome (LSE), (5) maturation of multivesicular bodies (MVBs), and formation of intraluminal vesicles (ILVs). (6) Fusion of ILVs with the plasma membrane and release of ILVs to the extracellular space as exosomes or (7) degradation by fusion with lysosomes or autophagosomes.

Therefore, MVBs are ILV-rich compartments that will eventually become exosomes upon release into the extracellular space. While there must be a fusion between the MVB and the plasma membrane to release the ILVs, there is also degradation by lysosome fusion or through autophagosomes (Figure 4) [56,57].

Exosomes’ cargo is not haphazardly distributed; instead, the load depends on the origin conditions [58]. Several cell types secrete circulating miRNAs, which can be found in various body fluids, such as saliva, urine, serum, and plasma [59,60]. The circulating miRNAs in serum and plasma can be obtained by extracting the total RNA from a sample and measuring it by real-time PCR [61]. This is because miRNAs are contained within exosomes or bound to proteins (high-density lipoprotein (HDL) and nucleophosmin 1), maintaining their integrity until released into the recipient cell [50,62,63].

The mechanism by which miRNAs are loaded into exosomes appears to depend on certain proteins, like neutral sphingomyelinase 2, and on the endosomal sorting complex required for transport (ESCRT), a protein complex involved in packaging biomolecules into the LVIs of MVBs [64]. Other proteins that play a role in the loading of miRNAs into exosomes are GW182-AGO2 proteins that are part of the miRNA effector complex, as well as RNA-binding proteins such as heterogeneous ribonucleoproteins-A2B, Y-box 1, and mex-3 RNA-binding family member C [65,66,67,68].

**Figure 5:**
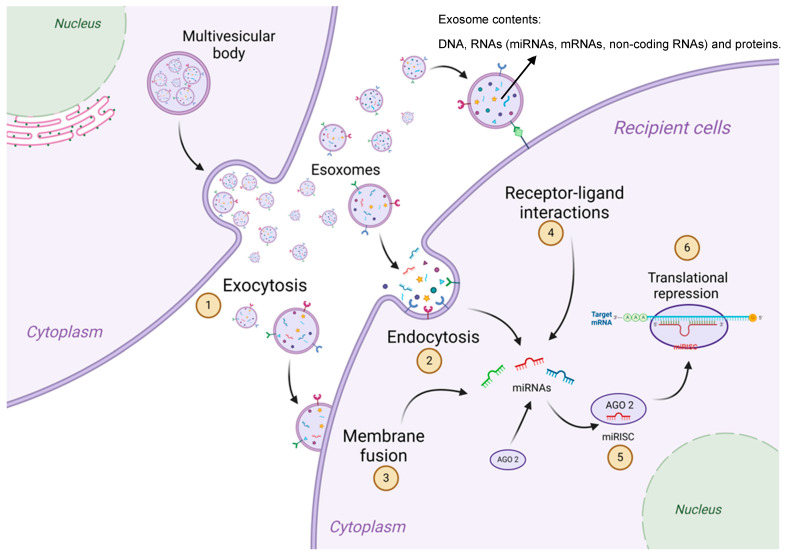
Schematic representation of the exosome release mechanisms. (1) Exosomes are released into the extracellular space and might deliver their contents such as DNA, RNAs (miRNAs, mRNAs, non-coding RNAs) and proteins to the recipient cell through (2) endocytosis, (3) membrane fusion between the exosome and the recipient cell, and (4) receptor–ligand interaction. (5 and 6) The miRNAs released from the exosome will continue in the miRNA pathway, binding to the AGO2 protein (miRISC), where the miRNA will be directed to the target mRNA.

MVBs can release exosomes through diverse mechanisms, mainly via fusion with the plasma membrane and subsequent release of the exosomes into the extracellular space. An additional pathway could be by exosomal binding and endocytosis of target cell receptors (Figure 5) [69].

## 7. Upregulated Serum/Plasma miRNAs in Idiopathic Pulmonary Fibrosis

### 7.1. miR-21

Many circulating miRNAs have been reported in IPF (serum/plasma); some are overexpressed and others are downregulated. miR-21 has been found to be augmented in serum/plasma samples from IPF patients, and it has been proposed as a predictor of the IPF prognosis [8,9,10]. Its target is SMAD7, an important protein in the TGF-β-1 signaling pathway, since it is an inhibitory protein of the SMAD2/3 [70,71]. This regulation is crucial because TGF-β1 signaling can trigger several molecular events in IPF, like increased expression of ECM proteins (collagen I type I, collagen III, etc.), induce fibroblast proliferation, EMT, etc. [72,73,74]. The TGF-β1 signaling pathway induces a positive loop because it increases the expression of miR-21. It has been reported that the SMADs (2,3,4) regulate the promoter activity of miR-21 [75]. miR-21 has been consistently observed to promote lung fibrosis in fibroblasts and AECs. Another target for miR-21 is the phosphatase and tensin homolog (PTEN) protein. Its inhibition induced EMT in a fibrosis model of ionizing radiation and senescence in AECs [76,77,78,79]. In lung fibroblasts, miR-21/PTEN decreases the effects of TGF-beta 1 mediated by the methylation of pri-miR-21 by MTTL3. PTEN is decreased in the serum of patients with IPF, and it seems to have a protective effect by decreasing proliferation, increasing apoptosis, and reducing the levels of α-SMA [80,81].

Resveratrol has been reported to be an antifibrotic agent, but, notwithstanding, miR-21 could reverse the effects of resveratrol in a bleomycin model in rats, and contrastingly, bleomycin treatment diminished the expression of miR-21 in rats [82].

### 7.2. miR-155

The levels of serum miR-155 in IPF are elevated in comparison with the control group [8,10]. miR-155 is related to the development of pulmonary fibrosis in several models in vitro and in vivo. It has been reported that miR-155 targets keratinocyte growth factor (KGF), which has been found to have a protective effect because, in the mouse model of bleomycin, animals instilled and treated with intravenous KGF showed a decrease in mortality and morbidity. Administration of viral vectors to overexpress KGF in mice instilled with bleomycin showed a reduction in the collagen I and TGF-β1 levels. Furthermore, in a model of hypoxia-induced fibrosis, miR155 was observed to promote EMT in HK-2 cells [83,84,85,86]. In addition, it has been observed that, in the silicosis model in mice, miR-155 shows profibrotic effects by the inhibition of mephrin α. This protein has antifibrotic effects because it decreases TGF-β1 expression and its receptors (TGFBRI and TGFBRII) [87]. The inhibition of miR-155 in an IPF bleomycin model resulted in diminished profibrotic effects that downregulated TGF-β1 and IL4 (interleukin 4) [88]. Similarly, the miR-155 plasmatic concentration is elevated in pulmonary fibrosis related to systemic sclerosis, since it is required for collagen synthesis during the fibrotic process [89]. It has been proposed that this mechanism is promoted by miR-155 inhibition of the protein forkhead box O3 (FOXO3a), resulting in the activation of the NLR family pyrin domain-containing 3 (NLRP3) [90]. However, evidence about the role of miR-155 in the development of pulmonary fibrosis remains contradictory, and it has been reported that miR-155 inhibits the epithelial–mesenchymal transition (EMT) by targeting the glycogen synthase 3 beta (GSK-3β) and its interaction with liver X receptor (LXR) to exert antifibrotic effects [91].

### 7.3. miR-590-3p

miR-590-3p is also augmented in the plasma of IPF patients, but no similar evidence has been found in animal models of pulmonary fibrosis. However, reports in other tissues suggest that the effect of this miRNA depends on its location [11]. For example, in heart fibroblasts, miR-590-3p regulates proliferation, migration, and collagen synthesis by acting on the zinc finger E-box-binding homeobox 1 (ZEB1) protein [92]. In contrast, miR-590-3p inhibits the mouse double minute 2 homolog (MDM2) protein in hepatocellular carcinoma, inhibiting EMT [93]. The effect of miR-590-3p in the development of pulmonary fibrosis is not clear. Perhaps it depends on its location, as has been reported for other molecules in IPF, like matrix metalloproteinase 1 (MMP1). MMP1 is overexpressed in epithelial cells of IPF, but not in lung fibroblasts, where it is diminished [94]. This intriguing issue clearly deserves further and more detailed research.

### 7.4. miR-199a-5p and miR-200c

miR-199a-5p has the most consistent results because it was the first miRNA to be reported to be elevated in a bleomycin model of lung fibrosis and tissues from IPF patients. Concordantly, the serum miR-199a-5p levels are elevated in IPF and can be used as progression markers [12]. miR-199a-5p is elevated in the mouse bleomycin model, and its reported targets are caveolin-1 and sestrin-2 (SESN2). Caveolin-1 is expressed in AECs and lung fibroblasts; loss of caveolin-1 expression has been associated with lung fibrogenesis, and its levels are downregulated in the lung tissue of IPF patients. It has been reported that caveolin-1 has a protective role in IPF because it can inhibit apoptosis and decrease the expression levels of collagen I and α-smooth muscle actin [95,96,97,98,99,100,101,102]. SESN2 has been reported to have an antifibrotic effect in an IPF model induced by eupatilin; TGFβ1 reduces the SESN2 protein, sestrin2 [103]. In addition, miR-199a-5p has been related to the regulation of mesenchymal stem cell senescence in IPF [104]. However, the inverse effect was reported in liver fibrosis, in which the inhibition of miR-199a-5p diminished fibrosis (antifibrotic effect) [105].

miR-199a-5p targets activating transcription factor 6 α (ATF6α) and inositol-requiring enzyme 1 (IRE1); both have been reported to be sensors of endoplasmic reticulum stress (ERS) [106,107]. The levels of ATF6α and IRE1 are elevated in lung fibrosis (bleomycin and silica models) and in IPF tissues, which is contradictory according to the elevated levels of miR-199a-5p. However, in mice models of cardiac fibrosis, the absence of ATF6α in cardiac fibroblasts contributes to the increase of profibrotic fibroblast markers like collagen 1 and alpha-smooth muscle actin in mice [108].

On the other hand, miR-200c is elevated in serum from IPF patients and is associated with interstitial lung abnormalities [12,109]. In contrast, miR-200c is reduced in the lung of the bleomycin IPF model and in epithelial cells after treatment with TGF-β1, and the diminution of miR-200c was related to the development of EMT and loss of the ability of alveolar epithelial cells type II to differentiate into alveolar epithelial cells type I [110,111,112]. The process correlates with another target of miR-200c, cadherin 11 (CDH11), which is elevated in lung fibrosis and is related to fibroblast migration, myofibroblast differentiation, and the EMT process during lung injury [113,114,115]. Noteworthily, reduced miR-200c expression is relevant because it also regulates the fibronectin amounts, an MEC component that accumulates in the lung fibrotic process [116,117]. miR-200c seems to participate in regulating EMT through ZEB1 [118]. These examples suggest that miR-200c levels are downregulated in IPF.

## 8. Downregulated Serum/Plasma miRNAs in Idiopathic Pulmonary Fibrosis

### 8.1. Let-7a and Let-7d

Let-7 (lethal-7) miRNAs are a family of 10 members that include let-7a, let-7b, let-7c, let-7d, let-7e, let-7f, let-7g, let-7i, miR-98 and miR-202 [119]. Let-7a and Let-7d are downregulated in serum samples from patients with IPF and regulate several targets related to the development of lung fibrosis via the TGF-β1 signaling pathway. TGF-β1 is a profibrotic molecule that triggers various molecular events in the development of pulmonary fibrosis. Such events affect both alveolar epithelial cells and lung fibroblasts [12,120]. In the case of epithelial cells, TGF-β1 induces EMT, and activation of AECs results in the production of many profibrotic molecules, like plasminogen activator inhibitor 1 (PAI-1), CTGF, PDGF, etc. [74,121,122,123]. Lung pulmonary fibroblasts affected by the TGF-β1 signaling pathway are activated, which causes an increase in the expression levels of type I and III collagen and induces proliferation and fibroblast differentiation into myofibroblast (augmentation of α-smooth muscle actin), contributing to more aggressive collagen deposition and providing resistance to apoptosis [124,125,126,127,128,129,130,131].

The let-7a and let-7d target is the high-mobility group AT-hook 2 (HMGA2) protein, a chromatin protein that regulates gene transcription, participating as a co-factor for other transcription factors. It has been reported that let-7a and let-7d could suppress EMT and fibroblast proliferation by blocking HMGA2 and transfection of let-7d into lung fibroblasts, decreasing α-SMA expression, reducing proliferation and migration, and decreasing the HMGA2 levels that TGF-β1-induced [12,132,133,134,135]. In the same signaling pathway, the targets of let-7a and let-7d are the receptors of TGF-β1 (TGFBR1 and TGFBR3) [136,137,138]. According to Tarbase 9.0 (a database used to deliver experimentally supported miRNA targets on RNAm from AGO-CLIP-seq protocols), both miRNAs regulate SMAD2, SMAD3, and histone acetyltransferase CREBBP. This protein has been related to promoting an increase in the expression of collagen VI, which is augmented in lung fibrosis and participates in EMT, an important process in developing IPF [139,140,141,142].

Let-7a and Let-7d have other profibrotic targets, like the insulin-like growth factor receptor (IGF1R) and the platelet-derived growth factor B (PDGFB), and, interestingly, receptors of these both profibrotic targets are overexpressed in IPF. Another profibrotic target of let-7a is the activin A receptor type 1B (ACVR1B), which has been implicated in the pathogenesis of cardiac fibrosis through activation by activin A. Human lung fibroblasts express the receptors of activin A (ACVR1B included) and contribute to a profibrotic microenvironment by promoting collagen contraction, a crucial event in lung fibrosis development [123,143,144,145,146].

### 8.2. miR-16

Serum miR-16 is downregulated in IPF, and this miRNA has antifibrotic activity [13]. miR-16 inhibits the activity of TGF-β1 and collagen I expression in dermal fibroblasts, and this inhibition is attributed to miR-16 targeting SMAD 2 and SMAD3 [147,148]. In hepatic stellate cells, miR-16 suppresses the proliferation and fibrogenesis provoked by TGF-β1 regulating lysyl oxidase homolog 1 (LOX L1) [149]. Other evidence of the antifibrotic effects of miR-16 can be found in systemic sclerosis, where it suppresses myofibroblast activation by inhibiting neurogenic locus homolog protein 2 (NOTCH2) [150]. HMGA1 and HMGA2, fibroblast growth factor receptor 1 (FGFR-1) and IGF1R also are targets for miR-16 [151,152,153].

### 8.3. miR-25-3p and miR-142-5p

miR-25-3p is downregulated in pulmonary fibrosis, but there are no data about its role in lung fibrosis. It has been reported that its expression is increased in cardiac fibrosis and related to the augmentation of α-smooth muscle actin [14,154]. In contrast, in hepatic stellate cells, the overexpression of miR-25-3p has a positive effect because it favors the Notch 1 signaling pathway, inducing profibrotic results via collagen expression through TGF-β1 [155]. Additionally, there is a report mentioning that miR-25-3p inhibition indirectly caused the elevation of TGF-β1, while another report stated that SMAD2 was a target for miR-25-3p. These findings reinforce the relationship between miR-25-3p and the TGF-β1 signaling pathway [156,157]. miR-142-5p is downregulated in IPF serum, and reports of hepatic fibrosis have linked it to an antifibrotic effect since, when overexpressed, hepatic fibrosis decreases. Interestingly, it has been found to be increased in esophageal fibrosis [10,158,159]. In the same sense, the nuclear factor erythroid 2-related factor 2 (Nrf2) transcription factor has been reported to be an miR-142- 5p target; this transcription factor is considered to be antifibrotic because it negatively regulates the TGF-β1 signaling pathway [160,161,162].

### 8.4. miR-101-3p

miR-101-3p is downregulated in IPF serum and lung tissue. This is important because miR-101-3p has been reported to regulate the WNT5a signaling pathway, which reduces proliferation in lung fibroblasts by regulating the receptor frizzled-4,6 (FZD4/6). WNT5a is elevated in IPF, and its signaling pathway has many profibrotic effects, such as inducing fibroblast proliferation, increasing resistance to apoptosis, and promoting fibronectin expression. miR-101-3p also targets the TGFBRI receptor of the TGF-β1 signaling pathway, and it seems that miR101-3p regulates both the WNT5a and TGFβ1 signaling pathways in a coordinated manner [10,163,164,165,166]. Another target of miR-101 with profibrotic effects is endothelin-1; this protein is elevated in the serum of patients with IPF and has many profibrotic effects, like inducing the production of collagen I and III, as well as the overproduction of CTGF, fibronectin, α-smooth muscle actin [167,168,169,170].

## 9. Discussion

**Figure 6:**
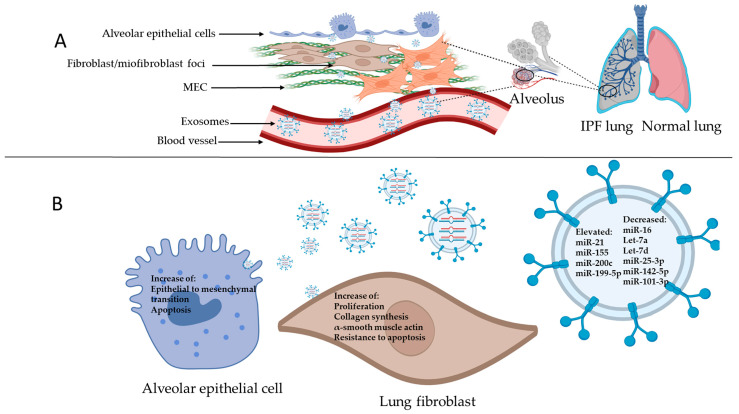
(**A**) Figure representing an IPF alveolus, where there is excessive fibroblast proliferation and exaggerated accumulation of ECM, EMT, and exosomes with their miRNAs. (**B**) Exosomes are incorporated into the cells in the profibrotic microenvironment (AECs and fibroblasts), resulting in an increase in molecular processes within the IPF, such as an increase apoptosis and EMT (AECs), and an increase in fibroblast proliferation, collagen synthesis, resistance to apoptosis and their differentiation into myofibroblasts (increase in α-smooth muscle actin).

Serum/plasma biomarkers in IPF have been investigated for a long time (Figure 6) [171]. In this regard, let-7a and let-7d are generally downregulated, and miR-21 is elevated. The results obtained in serum and lung fibroblast studies are consistent when compared to other miRNAs evaluated in serum/plasma from IPF patients. In this context, the miR-21 levels have been found to be associated with some clinical and radiological variables of patients with IPF, such as the FVC, DLCO, and HRCT, in addition to being related to the development of EMT (analyzed in the bleomycin model) [8,10,11]. Therefore, miR-21 has been suggested as an interesting therapeutic target, as reported by Lingyue Yan et al., where the release of an anti-miR-21 through liposomes, both in vitro (lung fibroblasts) and in vivo (bleomycin model), resulted in the decrease of pro-fibrosing markers such as fibronectin 1, collagen 1 and α-SMA [172]. In a similar way to what was reported by Gang Liu et al. in 2010, it was observed that miR-21 was a mediator of profibrotic activation in lung fibroblasts [70].

Let-7a is decreased in IPF, but interestingly, this decrease is greater in patients with rapidly progressive IPF compared to slowly progressive IPF [12]. In the case of let-7d, its decrease is related to the increase in the profibrotic profile of lung fibroblasts and the increase in EMT [132,135]. It has been suggested that this increase in EMT is due to the overexpression of the lncRNA Lin28B that induces the reduction of let-7d levels, which is reversed when miR-26a regulates lin28B [173].

**Figure 7:**
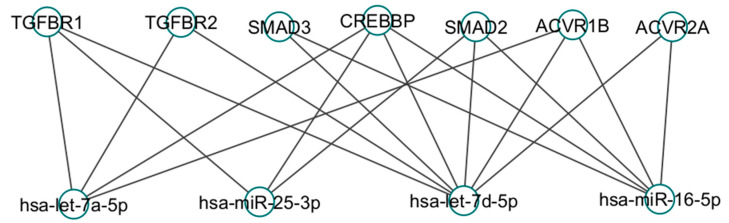
Four miRNAs have been found to be decreased in the serum of patients with IPF and to share target messenger RNAs that play an important role in IPF.

However, data indicate that some miRNAs that are decreased in the serum of IPF patients, such as let-7d, let-7a, miR-16-5p and miR-25-3p, target several components of the TGF-β1 signaling pathway. These data are summarized in Figure 7 [8,9,10,12,75,132].

The importance of finding noninvasive biomarkers is due to the imperative necessity of accurately distinguishing between IPF and other PF-ILDs with a UIP pattern (like hypersensitivity pneumonitis and rheumatoid arthritis), and consequently, to establish a prompt differential diagnosis and treatment [4,7,174]. miRNAs have emerged as a promising molecule that could be evaluated in the serum/plasma of IPF patients and fulfill these expectancies [8,50]. Because miRNAs are able to move through the bloodstream protected by exosomes and with protein Argonaute 2 complexes and later be detected by their isolation through total RNA extraction and its subsequent real-time PCR evaluation, they are now considered to possess the uttermost diagnostic potential. Notwithstanding the serum/plasma in ILDs, the miRNAs in IPF patients have only been evaluated compared with the control group [175,176]. In this regard, let-7a and let-7d are generally downregulated, and miR-21 is elevated. The results obtained in serum and lung fibroblast studies are consistent when compared to other miRNAs evaluated in serum/plasma from IPF patients. But today, there is no report that compares the serum/plasma miRNAs levels between hypersensitivity pneumonitis and IPF to find some miRNAs that are differentially expressed. There is only one report in fibroblasts [177].

Understandably, the goal of miRNAs studies in IPF serum or plasma is to correlate their expression levels with the clinical characteristics of the illness to find out if they are reliable biomarkers that could serve as diagnostic or prognostic tools [178,179]. Additional information obtained indirectly from the serum/plasma miRNAs profile is used to link the signaling pathways in which they are involved through the in silico analysis of the reported target mRNAs [180]. In addition, the serum miRNA analysis might also point out new signaling pathways that could participate in the pathogenesis of IPF, knowledge that can be extended by using software that predicts new targets of this type of RNA [181].

During the writing of this narrative review, we found that there are miRNAs with dual functions within IPF, such as miR-590-3p and miR-200c, including miR-155, which are elevated in IPF serum [8,11,12]. But the findings reported in IPF tissue, lung fibroblasts, or in the bleomycin model are contradictory when compared to each other. For example, in the miR-590-3p section, we suggest that this may be due to a phenomenon similar to what happens with MMP1 in the IPF [94]. The above was also discussed in a review by Zhimin Zou et al., where they suggest that the different levels of miR-590-3p are due to a self-regulatory feedback loop with TGF-beta1 and that it would depend on the stage of the fibrotic process [182].

On the other hand, the contradictory results regarding the miRNAs levels in serum/plasma compared with their levels in tissue and fibroblasts from patients with IPF could be explained by the regulatory effect of long non-coding RNAs that have the function of limiting miRNAs [183]. In addition, miRNAs are subjected to the RNA-editing mechanism carried out by ADAR proteins. This process could affect the rate of miRNA processing and redirect them to another target mRNA, a process that until now has been little studied within the mechanisms of action of miRNAs [184].

## 10. Conclusions

Given the complexity of the diagnosis of IPF due to the clinical characteristics that it shares with other ILDs, the search for biomarkers within this type of fibrosing lung diseases is essential. Finding biomarkers that could allow clinicians to differentiate IPF from other ILDs would constitute an invaluable diagnostic tool, because IPF is the interstitial lung disease with the highest incidence and the worst prognosis. Measuring miRNAs’ amounts in plasma or serum has opened up new possibilities to achieve this objective. In this sense, recent findings point out that the expression levels of several miRNAs in serum/plasma coincide with those reported in in vitro and in vivo models of IPF (let-7d, let-7a, miR21). But the expression levels of other miRNAs, such as miR-155, miR-199a-5p, and miR-200c, showed contradictory results; where some reports associated them with profibrotic activity, others claimed to have seen antifibrotic activity. Based on all the information analyzed in this narrative review, it is necessary to compare the measurements in serum/plasma IPF with other ILDs to refine the results obtained until now.

## 11. Limitations and Future Research

One limitation of this review was that it did not include a section regarding long non-coding RNAs as part of the regulation of miRNAs, since these are also measured in the serum/plasma of patients with IPF. However, the search for IPF biomarkers is essential due to the challenges involved in the diagnosis of ILDs, which require a multidisciplinary group with experience in their diagnosis. There is an imperative need to continue searching for new miRNAs in IPF and to be able to compare them with other ILDs such as fibrotic hypersensitivity pneumonitis and rheumatoid arthritis, illnesses that present fibrotic phases similar to IPF.

## Figures and Tables

**Table 1:** Summarizes relevant clinical information related to the miRNAs evaluated in this narrative review.

miRNAs in IPF	Sample Type (Serum/Plasma)	Relevant Information in the Cohort of the Study	References
miR-21-5p ↑	Serum/plasma	Predictor of the prognosis in IPF. Associated with degree of damage (FVC and HRCT). Associated with DLCO. More elevated in rapidly progressive IPF patients than in slowly progressive IPF patients.	[8,9,10]
miR-590-3p ↑	Plasma	Associated with DLCO.	[11]
miR-155 ↑	Serum	Correlated with radiologic examinations (HRCT). Associated with FVC.	[8,10]
miR-199-5p ↑	Serum	More elevated in rapidly progressive IPF patients than in slowly progressive IPF patients.	[12]
miR-200c ↑	Serum	Elevated in slowly and rapidly progressive IPF patients. Elevated in subjects with interstitial lung abnormalities (non-IPF or ILD patients).	[12]
Let 7a and Let 7d ↓	Serum/plasma	Let-7a downregulated in slowly and rapidly progressive IPF patients. Let-7d downregulated levels correlate with acute exacerbation of IPF. Let-7d is more downregulated in rapidly progressive IPF patients than in slowly progressive IPF patients.	[12,13]
miR-101-3p ↓	Serum	Associated with FVC.	[10]
miR-25-3p ↓	Plasma	Downregulated in stable IPF patients but increased in acute exacerbation IPF.	[14]

FVC = forced vital capacity, DLCO = carbon monoxide diffusing capacity, HRCT = high-resolution computed tomography. Up arrows (↑) increased expression, down arrows (↓) decreased expression.

## References

[1] Nho R.S. (2015). Alteration of Aging-Dependent MicroRNAs in Idiopathic Pulmonary Fibrosis. Drug Dev. Res..

[2] Guler S.A., Corte T.J. (2021). Interstitial Lung Disease in 2020: A History of Progress. Clin. Chest Med..

[3] Samarelli A.V., Tonelli R., Marchioni A., Bruzzi G., Gozzi F., Andrisani D., Castaniere I., Manicardi L., Moretti A., Tabbi L. (2021). Fibrotic Idiopathic Interstitial Lung Disease: The Molecular and Cellular Key Players. Int. J. Mol. Sci..

[4] Raghu G., Remy-Jardin M., Richeldi L., Thomson C.C., Inoue Y., Johkoh T., Kreuter M., Lynch D.A., Maher T.M., Martinez F.J. (2022). Idiopathic Pulmonary Fibrosis (an Update) and Progressive Pulmonary Fibrosis in Adults: An Official ATS/ERS/JRS/ALAT Clinical Practice Guideline. Am. J. Respir. Crit. Care Med..

[5] Selman M., Pardo A. (2021). When things go wrong: Exploring possible mechanisms driving the progressive fibrosis phenotype in interstitial lung diseases. Eur. Respir. J..

[6] Confalonieri P., Volpe M.C., Jacob J., Maiocchi S., Salton F., Ruaro B., Confalonieri M., Braga L. (2022). Regeneration or Repair? The Role of Alveolar Epithelial Cells in the Pathogenesis of Idiopathic Pulmonary Fibrosis (IPF). Cells.

[7] Selman M., Pardo A., Wells A.U. (2023). Usual interstitial pneumonia as a stand-alone diagnostic entity: The case for a paradigm shift?. Lancet Respir. Med..

[8] Li P., Zhao G.Q., Chen T.F., Chang J.X., Wang H.Q., Chen S.S., Zhang G.J. (2013). Serum miR-21 and miR-155 expression in idiopathic pulmonary fibrosis. J. Asthma.

[9] Makiguchi T., Yamada M., Yoshioka Y., Sugiura H., Koarai A., Chiba S., Fujino N., Tojo Y., Ota C., Kubo H. (2016). Serum extracellular vesicular miR-21-5p is a predictor of the prognosis in idiopathic pulmonary fibrosis. Respir. Res..

[10] Li P., Li J., Chen T., Wang H., Chu H., Chang J., Zang W., Wang Y., Ma Y., Du Y. (2014). Expression analysis of serum microRNAs in idiopathic pulmonary fibrosis. Int. J. Mol. Med..

[11] Dirol H., Toylu A., Ogus A.C., Cilli A., Ozbudak O., Clark O.A., Ozdemir T. (2022). Alterations in plasma miR-21, miR-590, miR-192 and miR-215 in idiopathic pulmonary fibrosis and their clinical importance. Mol. Biol. Rep..

[12] Yang G., Yang L., Wang W., Wang J., Wang J., Xu Z. (2015). Discovery and validation of extracellular/circulating microRNAs during idiopathic pulmonary fibrosis disease progression. Gene.

[13] Lacedonia D., Scioscia G., Soccio P., Conese M., Catucci L., Palladino G.P., Simone F., Quarato C.M.I., Di Gioia S., Rana R. (2021). Downregulation of exosomal let-7d and miR-16 in idiopathic pulmonary fibrosis. BMC Pulm. Med..

[14] Min H., Fan S., Song S., Zhuang Y., Li H., Wu Y., Cai H., Yi L., Dai J., Gao Q. (2016). Plasma microRNAs are associated with acute exacerbation in idiopathic pulmonary fibrosis. Diagn. Pathol..

[15] Selman M., Pardo A. (2006). Role of epithelial cells in idiopathic pulmonary fibrosis: From innocent targets to serial killers. Proc. Am. Thorac. Soc..

[16] Yang J., Velikoff M., Canalis E., Horowitz J.C., Kim K.K. (2014). Activated alveolar epithelial cells initiate fibrosis through autocrine and paracrine secretion of connective tissue growth factor. Am. J. Physiol. Lung Cell. Mol. Physiol..

[17] Antoniades H.N., Bravo M.A., Avila R.E., Galanopoulos T., Neville-Golden J., Maxwell M., Selman M. (1990). Platelet-derived growth factor in idiopathic pulmonary fibrosis. J. Clin. Investig..

[18] Selman M., Pardo A. (2020). The leading role of epithelial cells in the pathogenesis of idiopathic pulmonary fibrosis. Cell Signal..

[19] Selman M., King T.E., Pardo A., American Thoracic S., European Respiratory S., American College of Chest P. (2001). Idiopathic pulmonary fibrosis: Prevailing and evolving hypotheses about its pathogenesis and implications for therapy. Ann. Intern. Med..

[20] Martinez F.J., Collard H.R., Pardo A., Raghu G., Richeldi L., Selman M., Swigris J.J., Taniguchi H., Wells A.U. (2017). Idiopathic pulmonary fibrosis. Nat. Rev. Dis. Primers.

[21] Pardo A., Selman M. (2021). The Interplay of the Genetic Architecture, Aging, and Environmental Factors in the Pathogenesis of Idiopathic Pulmonary Fibrosis. Am. J. Respir. Cell Mol. Biol..

[22] Kishaba T. (2019). Acute Exacerbation of Idiopathic Pulmonary Fibrosis. Medicina.

[23] Natsuizaka M., Chiba H., Kuronuma K., Otsuka M., Kudo K., Mori M., Bando M., Sugiyama Y., Takahashi H. (2014). Epidemiologic survey of Japanese patients with idiopathic pulmonary fibrosis and investigation of ethnic differences. Am. J. Respir. Crit. Care Med..

[24] Raghu G., Amatto V.C., Behr J., Stowasser S. (2015). Comorbidities in idiopathic pulmonary fibrosis patients: A systematic literature review. Eur. Respir. J..

[25] Gonzalez-Garcia M., Rincon-Alvarez E., Alberti M.L., Duran M., Caro F., Venero M.D.C., Liberato Y.E., Buendia-Roldan I. (2021). Comorbidities of Patients With Idiopathic Pulmonary Fibrosis in Four Latin American Countries. Are There Differences by Country and Altitude?. Front. Med..

[26] Cobra S.B., Rodrigues M.P., de Melo F.X., Ferreira N.M.C., Passini V.V., Amado V.M., Melo-Silva C.A. (2021). Right ventricular dysfunction in patients with non-severe idiopathic pulmonary fibrosis: A cross-sectional prospective single-center study. Expert Rev. Respir. Med..

[27] Buonauro A., Santoro C., Galderisi M., Canora A., Sorrentino R., Esposito R., Lembo M., Canonico M.E., Ilardi F., Fazio V. (2020). Impaired Right and Left Ventricular Longitudinal Function in Patients with Fibrotic Interstitial Lung Diseases. J. Clin. Med..

[28] Boon K., Bailey N.W., Yang J., Steel M.P., Groshong S., Kervitsky D., Brown K.K., Schwarz M.I., Schwartz D.A. (2009). Molecular phenotypes distinguish patients with relatively stable from progressive idiopathic pulmonary fibrosis (IPF). PLoS ONE.

[29] Rosas I.O., Richards T.J., Konishi K., Zhang Y., Gibson K., Lokshin A.E., Lindell K.O., Cisneros J., Macdonald S.D., Pardo A. (2008). MMP1 and MMP7 as potential peripheral blood biomarkers in idiopathic pulmonary fibrosis. PLoS Med..

[30] Greene K.E., King T.E., Kuroki Y., Bucher-Bartelson B., Hunninghake G.W., Newman L.S., Nagae H., Mason R.J. (2002). Serum surfactant proteins-A and -D as biomarkers in idiopathic pulmonary fibrosis. Eur. Respir. J..

[31] Hamai K., Iwamoto H., Ishikawa N., Horimasu Y., Masuda T., Miyamoto S., Nakashima T., Ohshimo S., Fujitaka K., Hamada H. (2016). Comparative Study of Circulating MMP-7, CCL18, KL-6, SP-A, and SP-D as Disease Markers of Idiopathic Pulmonary Fibrosis. Dis. Markers.

[32] Eduardo M., Ivette B.R., Gabriela D.P., Veronica M.A., Victor R. (2019). Evaluation of Renin and Soluble (Pro)renin Receptor in Patients with IPF. A Comparison with Hypersensitivity Pneumonitis. Lung.

[33] Rodriguez A., Griffiths-Jones S., Ashurst J.L., Bradley A. (2004). Identification of mammalian microRNA host genes and transcription units. Genome Res..

[34] Lee Y., Kim M., Han J., Yeom K.H., Lee S., Baek S.H., Kim V.N. (2004). MicroRNA genes are transcribed by RNA polymerase II. EMBO J..

[35] Lee Y., Jeon K., Lee J.T., Kim S., Kim V.N. (2002). MicroRNA maturation: Stepwise processing and subcellular localization. EMBO J..

[36] Lee Y., Ahn C., Han J., Choi H., Kim J., Yim J., Lee J., Provost P., Radmark O., Kim S. (2003). The nuclear RNase III Drosha initiates microRNA processing. Nature.

[37] Gregory R.I., Yan K.P., Amuthan G., Chendrimada T., Doratotaj B., Cooch N., Shiekhattar R. (2004). The Microprocessor complex mediates the genesis of microRNAs. Nature.

[38] Bohnsack M.T., Czaplinski K., Gorlich D. (2004). Exportin 5 is a RanGTP-dependent dsRNA-binding protein that mediates nuclear export of pre-miRNAs. RNA.

[39] Yi R., Qin Y., Macara I.G., Cullen B.R. (2003). Exportin-5 mediates the nuclear export of pre-microRNAs and short hairpin RNAs. Genes Dev..

[40] Gregory R.I., Chendrimada T.P., Cooch N., Shiekhattar R. (2005). Human RISC couples microRNA biogenesis and posttranscriptional gene silencing. Cell.

[41] Takimoto K., Wakiyama M., Yokoyama S. (2009). Mammalian GW182 contains multiple Argonaute-binding sites and functions in microRNA-mediated translational repression. RNA.

[42] Fabian M.R., Sonenberg N. (2012). The mechanics of miRNA-mediated gene silencing: A look under the hood of miRISC. Nat. Struct. Mol. Biol..

[43] Chendrimada T.P., Gregory R.I., Kumaraswamy E., Norman J., Cooch N., Nishikura K., Shiekhattar R. (2005). TRBP recruits the Dicer complex to Ago2 for microRNA processing and gene silencing. Nature.

[44] Haase A.D., Jaskiewicz L., Zhang H., Laine S., Sack R., Gatignol A., Filipowicz W. (2005). TRBP, a regulator of cellular PKR and HIV-1 virus expression, interacts with Dicer and functions in RNA silencing. EMBO Rep..

[45] Tomari Y., Matranga C., Haley B., Martinez N., Zamore P.D. (2004). A protein sensor for siRNA asymmetry. Science.

[46] Eulalio A., Huntzinger E., Izaurralde E. (2008). Getting to the root of miRNA-mediated gene silencing. Cell.

[47] Chevillet J.R., Kang Q., Ruf I.K., Briggs H.A., Vojtech L.N., Hughes S.M., Cheng H.H., Arroyo J.D., Meredith E.K., Gallichotte E.N. (2014). Quantitative and stoichiometric analysis of the microRNA content of exosomes. Proc. Natl. Acad. Sci. USA.

[48] Lasser C., Alikhani V.S., Ekstrom K., Eldh M., Paredes P.T., Bossios A., Sjostrand M., Gabrielsson S., Lotvall J., Valadi H. (2011). Human saliva, plasma and breast milk exosomes contain RNA: Uptake by macrophages. J. Transl. Med..

[49] Jeppesen D.K., Fenix A.M., Franklin J.L., Higginbotham J.N., Zhang Q., Zimmerman L.J., Liebler D.C., Ping J., Liu Q., Evans R. (2019). Reassessment of Exosome Composition. Cell.

[50] Valadi H., Ekstrom K., Bossios A., Sjostrand M., Lee J.J., Lotvall J.O. (2007). Exosome-mediated transfer of mRNAs and microRNAs is a novel mechanism of genetic exchange between cells. Nat. Cell Biol..

[51] Haraszti R.A., Didiot M.C., Sapp E., Leszyk J., Shaffer S.A., Rockwell H.E., Gao F., Narain N.R., DiFiglia M., Kiebish M.A. (2016). High-resolution proteomic and lipidomic analysis of exosomes and microvesicles from different cell sources. J. Extracell. Vesicles.

[52] Thery C., Zitvogel L., Amigorena S. (2002). Exosomes: Composition, biogenesis and function. Nat. Rev. Immunol..

[53] Woodman P.G., Futter C.E. (2008). Multivesicular bodies: Co-ordinated progression to maturity. Curr. Opin. Cell Biol..

[54] Grant B.D., Donaldson J.G. (2009). Pathways and mechanisms of endocytic recycling. Nat. Rev. Mol. Cell Biol..

[55] Stuffers S., Sem Wegner C., Stenmark H., Brech A. (2009). Multivesicular endosome biogenesis in the absence of ESCRTs. Traffic.

[56] Gruenberg J., van der Goot F.G. (2006). Mechanisms of pathogen entry through the endosomal compartments. Nat. Rev. Mol. Cell Biol..

[57] Williams R.L., Urbe S. (2007). The emerging shape of the ESCRT machinery. Nat. Rev. Mol. Cell Biol..

[58] Guduric-Fuchs J., O’Connor A., Camp B., O’Neill C.L., Medina R.J., Simpson D.A. (2012). Selective extracellular vesicle-mediated export of an overlapping set of microRNAs from multiple cell types. BMC Genom..

[59] Romani C., Baronchelli M., Assoni C., Mattavelli D., Calza S., Piazza C., Bossi P. (2024). Stability of circulating miRNA in saliva: The influence of sample associated pre-analytical variables. Clin. Chim. Acta.

[60] Elliot S., Catanuto P., Pereira-Simon S., Xia X., Shahzeidi S., Roberts E., Ludlow J., Hamdan S., Daunert S., Parra J. (2022). Urine-derived exosomes from individuals with IPF carry pro-fibrotic cargo. eLife.

[61] Shaker F., Razi S., Rezaei N. (2024). Circulating miRNA and circulating tumor DNA application as liquid biopsy markers in gastric cancer. Clin. Biochem..

[62] Vickers K.C., Palmisano B.T., Shoucri B.M., Shamburek R.D., Remaley A.T. (2011). MicroRNAs are transported in plasma and delivered to recipient cells by high-density lipoproteins. Nat. Cell Biol..

[63] Wang K., Zhang S., Weber J., Baxter D., Galas D.J. (2010). Export of microRNAs and microRNA-protective protein by mammalian cells. Nucleic Acids Res..

[64] Kosaka N., Iguchi H., Yoshioka Y., Takeshita F., Matsuki Y., Ochiya T. (2010). Secretory mechanisms and intercellular transfer of microRNAs in living cells. J. Biol. Chem..

[65] Villarroya-Beltri C., Gutierrez-Vazquez C., Sanchez-Cabo F., Perez-Hernandez D., Vazquez J., Martin-Cofreces N., Martinez-Herrera D.J., Pascual-Montano A., Mittelbrunn M., Sanchez-Madrid F. (2013). Sumoylated hnRNPA2B1 controls the sorting of miRNAs into exosomes through binding to specific motifs. Nat. Commun..

[66] Shurtleff M.J., Temoche-Diaz M.M., Karfilis K.V., Ri S., Schekman R. (2016). Y-box protein 1 is required to sort microRNAs into exosomes in cells and in a cell-free reaction. eLife.

[67] Lu P., Li H., Li N., Singh R.N., Bishop C.E., Chen X., Lu B. (2017). MEX3C interacts with adaptor-related protein complex 2 and involves in miR-451a exosomal sorting. PLoS ONE.

[68] Gibbings D.J., Ciaudo C., Erhardt M., Voinnet O. (2009). Multivesicular bodies associate with components of miRNA effector complexes and modulate miRNA activity. Nat. Cell Biol..

[69] Mathivanan S., Lim J.W., Tauro B.J., Ji H., Moritz R.L., Simpson R.J. (2010). Proteomics analysis of A33 immunoaffinity-purified exosomes released from the human colon tumor cell line LIM1215 reveals a tissue-specific protein signature. Mol. Cell. Proteom..

[70] Liu G., Friggeri A., Yang Y., Milosevic J., Ding Q., Thannickal V.J., Kaminski N., Abraham E. (2010). miR-21 mediates fibrogenic activation of pulmonary fibroblasts and lung fibrosis. J. Exp. Med..

[71] Hayashi H., Abdollah S., Qiu Y., Cai J., Xu Y.Y., Grinnell B.W., Richardson M.A., Topper J.N., Gimbrone M.A., Wrana J.L. (1997). The MAD-related protein Smad7 associates with the TGFbeta receptor and functions as an antagonist of TGFbeta signaling. Cell.

[72] Fine A., Goldstein R.H. (1987). The effect of transforming growth factor-beta on cell proliferation and collagen formation by lung fibroblasts. J. Biol. Chem..

[73] Xiao L., Du Y., Shen Y., He Y., Zhao H., Li Z. (2012). TGF-beta 1 induced fibroblast proliferation is mediated by the FGF-2/ERK pathway. Front. Biosci. Landmark Ed.

[74] Willis B.C., Liebler J.M., Luby-Phelps K., Nicholson A.G., Crandall E.D., du Bois R.M., Borok Z. (2005). Induction of epithelial-mesenchymal transition in alveolar epithelial cells by transforming growth factor-beta1: Potential role in idiopathic pulmonary fibrosis. Am. J. Pathol..

[75] Zhou J., Xu Q., Zhang Q., Wang Z., Guan S. (2018). A novel molecular mechanism of microRNA-21 inducing pulmonary fibrosis and human pulmonary fibroblast extracellular matrix through transforming growth factor beta1-mediated SMADs activation. J. Cell. Biochem..

[76] Yamada M., Kubo H., Ota C., Takahashi T., Tando Y., Suzuki T., Fujino N., Makiguchi T., Takagi K., Suzuki T. (2013). The increase of microRNA-21 during lung fibrosis and its contribution to epithelial-mesenchymal transition in pulmonary epithelial cells. Respir. Res..

[77] Liu Z., Liang X., Li X., Liu X., Zhu M., Gu Y., Zhou P. (2019). MiRNA-21 functions in ionizing radiation-induced epithelium-to-mesenchymal transition (EMT) by downregulating PTEN. Toxicol. Res..

[78] Wang P., Xiao T., Li J., Wang D., Sun J., Cheng C., Ma H., Xue J., Li Y., Zhang A. (2021). miR-21 in EVs from pulmonary epithelial cells promotes myofibroblast differentiation via glycolysis in arsenic-induced pulmonary fibrosis. Environ. Pollut..

[79] Tian Y., Li H., Qiu T., Dai J., Zhang Y., Chen J., Cai H. (2019). Loss of PTEN induces lung fibrosis via alveolar epithelial cell senescence depending on NF-kappaB activation. Aging Cell.

[80] Lu Y., Liu Z., Zhang Y., Wu X., Bian W., Shan S., Yang D., Ren T. (2023). METTL3-mediated m6A RNA methylation induces the differentiation of lung resident mesenchymal stem cells into myofibroblasts via the miR-21/PTEN pathway. Respir. Res..

[81] Xie B., Zheng G., Li H., Yao X., Hong R., Li R., Yue W., Chen Y. (2016). Effects of the tumor suppressor PTEN on the pathogenesis of idiopathic pulmonary fibrosis in Chinese patients. Mol. Med. Rep..

[82] Wang J., He F., Chen L., Li Q., Jin S., Zheng H., Lin J., Zhang H., Ma S., Mei J. (2018). Resveratrol inhibits pulmonary fibrosis by regulating miR-21 through MAPK/AP-1 pathways. Biomed. Pharmacother..

[83] Pottier N., Maurin T., Chevalier B., Puissegur M.P., Lebrigand K., Robbe-Sermesant K., Bertero T., Lino Cardenas C.L., Courcot E., Rios G. (2009). Identification of keratinocyte growth factor as a target of microRNA-155 in lung fibroblasts: Implication in epithelial-mesenchymal interactions. PLoS ONE.

[84] Finch P.W., Rubin J.S. (2004). Keratinocyte growth factor/fibroblast growth factor 7, a homeostatic factor with therapeutic potential for epithelial protection and repair. Adv. Cancer Res..

[85] Sakamoto S., Yazawa T., Baba Y., Sato H., Kanegae Y., Hirai T., Saito I., Goto T., Kurahashi K. (2011). Keratinocyte growth factor gene transduction ameliorates pulmonary fibrosis induced by bleomycin in mice. Am. J. Respir. Cell Mol. Biol..

[86] Xie S., Chen H., Li F., Wang S., Guo J. (2015). Hypoxia-induced microRNA-155 promotes fibrosis in proximal tubule cells. Mol. Med. Rep..

[87] Chen Y., Xu D., Yao J., Wei Z., Li S., Gao X., Cai W., Mao N., Jin F., Li Y. (2020). Inhibition of miR-155-5p Exerts Anti-Fibrotic Effects in Silicotic Mice by Regulating Meprin alpha. Mol. Ther. Nucleic Acids.

[88] Sun X., Kang Y., Xue S., Zou J., Xu J., Tang D., Qin H. (2021). In vivo therapeutic success of MicroRNA-155 antagomir in a mouse model of pulmonary fibrosis induced by bleomycin. Korean J. Intern. Med..

[89] Christmann R.B., Wooten A., Sampaio-Barros P., Borges C.L., Carvalho C.R., Kairalla R.A., Feghali-Bostwick C., Ziemek J., Mei Y., Goummih S. (2016). miR-155 in the progression of lung fibrosis in systemic sclerosis. Arthritis Res. Ther..

[90] Artlett C.M., Sassi-Gaha S., Hope J.L., Feghali-Bostwick C.A., Katsikis P.D. (2017). Mir-155 is overexpressed in systemic sclerosis fibroblasts and is required for NLRP3 inflammasome-mediated collagen synthesis during fibrosis. Arthritis Res. Ther..

[91] Wang D., Liu Z., Yan Z., Liang X., Liu X., Liu Y., Wang P., Bai C., Gu Y., Zhou P.K. (2021). MiRNA-155-5p inhibits epithelium-to-mesenchymal transition (EMT) by targeting GSK-3beta during radiation-induced pulmonary fibrosis. Arch. Biochem. Biophys..

[92] Yuan X., Pan J., Wen L., Gong B., Li J., Gao H., Tan W., Liang S., Zhang H., Wang X. (2020). MiR-590-3p regulates proliferation, migration and collagen synthesis of cardiac fibroblast by targeting ZEB1. J. Cell. Mol. Med..

[93] Youssef A.I., Khaled G.M., Amleh A. (2023). Functional role and epithelial to mesenchymal transition of the miR-590-3p/MDM2 axis in hepatocellular carcinoma. BMC Cancer.

[94] Pardo A., Cabrera S., Maldonado M., Selman M. (2016). Role of matrix metalloproteinases in the pathogenesis of idiopathic pulmonary fibrosis. Respir. Res..

[95] Kasper M., Reimann T., Hempel U., Wenzel K.W., Bierhaus A., Schuh D., Dimmer V., Haroske G., Muller M. (1998). Loss of caveolin expression in type I pneumocytes as an indicator of subcellular alterations during lung fibrogenesis. Histochem. Cell Biol..

[96] Wang X.M., Zhang Y., Kim H.P., Zhou Z., Feghali-Bostwick C.A., Liu F., Ifedigbo E., Xu X., Oury T.D., Kaminski N. (2006). Caveolin-1: A critical regulator of lung fibrosis in idiopathic pulmonary fibrosis. J. Exp. Med..

[97] Odajima N., Betsuyaku T., Nasuhara Y., Nishimura M. (2007). Loss of caveolin-1 in bronchiolization in lung fibrosis. J. Histochem. Cytochem..

[98] Lino Cardenas C.L., Henaoui I.S., Courcot E., Roderburg C., Cauffiez C., Aubert S., Copin M.C., Wallaert B., Glowacki F., Dewaeles E. (2013). miR-199a-5p Is upregulated during fibrogenic response to tissue injury and mediates TGFbeta-induced lung fibroblast activation by targeting caveolin-1. PLoS Genet..

[99] Yang M., Yin E., Xu Y., Liu Y., Li T., Dong Z., Tai W. (2022). CDKN2B antisense RNA 1 expression alleviates idiopathic pulmonary fibrosis by functioning as a competing endogenouse RNA through the miR-199a-5p/Sestrin-2 axis. Bioengineered.

[100] Shetty S., Idell S. (2023). Caveolin-1-Related Intervention for Fibrotic Lung Diseases. Cells.

[101] Tourkina E., Richard M., Gooz P., Bonner M., Pannu J., Harley R., Bernatchez P.N., Sessa W.C., Silver R.M., Hoffman S. (2008). Antifibrotic properties of caveolin-1 scaffolding domain in vitro and in vivo. Am. J. Physiol. Lung Cell. Mol. Physiol..

[102] Marudamuthu A.S., Bhandary Y.P., Fan L., Radhakrishnan V., MacKenzie B., Maier E., Shetty S.K., Nagaraja M.R., Gopu V., Tiwari N. (2019). Caveolin-1-derived peptide limits development of pulmonary fibrosis. Sci. Transl. Med..

[103] Gong H., Lyu X., Liu Y., Peng N., Tan S., Dong L., Zhang X. (2023). Eupatilin inhibits pulmonary fibrosis by activating Sestrin2/PI3K/Akt/mTOR dependent autophagy pathway. Life Sci..

[104] Shi L., Han Q., Hong Y., Li W., Gong G., Cui J., Mao M., Liang X., Hu B., Li X. (2021). Inhibition of miR-199a-5p rejuvenates aged mesenchymal stem cells derived from patients with idiopathic pulmonary fibrosis and improves their therapeutic efficacy in experimental pulmonary fibrosis. Stem. Cell Res. Ther..

[105] Ding R., Zheng J., Li N., Cheng Q., Zhu M., Wang Y., Zhou X., Zhang Z., Shi G. (2021). DZNep, an inhibitor of the histone methyltransferase EZH2, suppresses hepatic fibrosis through regulating miR-199a-5p/SOCS7 pathway. PeerJ.

[106] Dai B.H., Geng L., Wang Y., Sui C.J., Xie F., Shen R.X., Shen W.F., Yang J.M. (2013). microRNA-199a-5p protects hepatocytes from bile acid-induced sustained endoplasmic reticulum stress. Cell Death Dis..

[107] Lei Y., Yu H., Ding S., Liu H., Liu C., Fu R. (2024). Molecular mechanism of ATF6 in unfolded protein response and its role in disease. Heliyon.

[108] Stauffer W.T., Blackwood E.A., Azizi K., Kaufman R.J., Glembotski C.C. (2020). The ER Unfolded Protein Response Effector, ATF6, Reduces Cardiac Fibrosis and Decreases Activation of Cardiac Fibroblasts. Int. J. Mol. Sci..

[109] Ortiz-Quintero B., Buendia-Roldan I., Ramirez-Salazar E.G., Balderas-Martinez Y.I., Ramirez-Rodriguez S.L., Martinez-Espinosa K., Selman M. (2020). Circulating microRNA Signature Associated to Interstitial Lung Abnormalities in Respiratory Asymptomatic Subjects. Cells.

[110] Yang S., Banerjee S., de Freitas A., Sanders Y.Y., Ding Q., Matalon S., Thannickal V.J., Abraham E., Liu G. (2012). Participation of miR-200 in pulmonary fibrosis. Am. J. Pathol..

[111] Liu Y., Li Y., Xu Q., Yao W., Wu Q., Yuan J., Yan W., Xu T., Ji X., Ni C. (2018). Long non-coding RNA-ATB promotes EMT during silica-induced pulmonary fibrosis by competitively binding miR-200c. Biochim. Biophys. Acta Mol. Basis Dis..

[112] Moimas S., Salton F., Kosmider B., Ring N., Volpe M.C., Bahmed K., Braga L., Rehman M., Vodret S., Graziani M.L. (2019). miR-200 family members reduce senescence and restore idiopathic pulmonary fibrosis type II alveolar epithelial cell transdifferentiation. ERJ Open Res..

[113] Luo D., Wilson J.M., Harvel N., Liu J., Pei L., Huang S., Hawthorn L., Shi H. (2013). A systematic evaluation of miRNA:mRNA interactions involved in the migration and invasion of breast cancer cells. J. Transl. Med..

[114] Schneider D.J., Wu M., Le T.T., Cho S.H., Brenner M.B., Blackburn M.R., Agarwal S.K. (2012). Cadherin-11 contributes to pulmonary fibrosis: Potential role in TGF-beta production and epithelial to mesenchymal transition. FASEB J..

[115] Black M., Milewski D., Le T., Ren X., Xu Y., Kalinichenko V.V., Kalin T.V. (2018). FOXF1 Inhibits Pulmonary Fibrosis by Preventing CDH2-CDH11 Cadherin Switch in Myofibroblasts. Cell Rep..

[116] Howe E.N., Cochrane D.R., Richer J.K. (2011). Targets of miR-200c mediate suppression of cell motility and anoikis resistance. Breast Cancer Res..

[117] Rennard S.I., Crystal R.G. (1982). Fibronectin in human bronchopulmonary lavage fluid. Elevation in patients with interstitial lung disease. J. Clin. Investig..

[118] Siemens H., Jackstadt R., Hunten S., Kaller M., Menssen A., Gotz U., Hermeking H. (2011). miR-34 and SNAIL form a double-negative feedback loop to regulate epithelial-mesenchymal transitions. Cell Cycle.

[119] Thammaiah C.K., Jayaram S. (2016). Role of let-7 family microRNA in breast cancer. Noncoding RNA Res..

[120] Ye Z., Hu Y. (2021). TGFbeta1: Gentlemanly orchestrator in idiopathic pulmonary fibrosis (Review). Int. J. Mol. Med..

[121] Shi L., Dong N., Fang X., Wang X. (2016). Regulatory mechanisms of TGF-beta1-induced fibrogenesis of human alveolar epithelial cells. J. Cell. Mol. Med..

[122] Rana T., Jiang C., Liu G., Miyata T., Antony V., Thannickal V.J., Liu R.M. (2020). PAI-1 Regulation of TGF-beta1-induced Alveolar Type II Cell Senescence, SASP Secretion, and SASP-mediated Activation of Alveolar Macrophages. Am. J. Respir. Cell Mol. Biol..

[123] Kasai H., Allen J.T., Mason R.M., Kamimura T., Zhang Z. (2005). TGF-beta1 induces human alveolar epithelial to mesenchymal cell transition (EMT). Respir. Res..

[124] Heine U.I., Munoz E.F., Flanders K.C., Roberts A.B., Sporn M.B. (1990). Colocalization of TGF-beta 1 and collagen I and III, fibronectin and glycosaminoglycans during lung branching morphogenesis. Development.

[125] Grande J.P., Melder D.C., Zinsmeister A.R. (1997). Modulation of collagen gene expression by cytokines: Stimulatory effect of transforming growth factor-beta1, with divergent effects of epidermal growth factor and tumor necrosis factor-alpha on collagen type I and collagen type IV. J. Lab. Clin. Med..

[126] Kenyon N.J., Ward R.W., McGrew G., Last J.A. (2003). TGF-beta1 causes airway fibrosis and increased collagen I and III mRNA in mice. Thorax.

[127] Krupsky M., Fine A., Kuang P.P., Berk J.L., Goldstein R.H. (1996). Regulation of type I collagen production by insulin and transforming growth factor-beta in human lung fibroblasts. Connect. Tissue Res..

[128] Raghu G., Masta S., Meyers D., Narayanan A.S. (1989). Collagen synthesis by normal and fibrotic human lung fibroblasts and the effect of transforming growth factor-beta. Am. Rev. Respir. Dis..

[129] Desmouliere A., Geinoz A., Gabbiani F., Gabbiani G. (1993). Transforming growth factor-beta 1 induces alpha-smooth muscle actin expression in granulation tissue myofibroblasts and in quiescent and growing cultured fibroblasts. J. Cell Biol..

[130] Zhang H.Y., Phan S.H. (1999). Inhibition of myofibroblast apoptosis by transforming growth factor beta(1). Am. J. Respir. Cell Mol. Biol..

[131] Montesano R., Orci L. (1988). Transforming growth factor beta stimulates collagen-matrix contraction by fibroblasts: Implications for wound healing. Proc. Natl. Acad. Sci. USA.

[132] Pandit K.V., Corcoran D., Yousef H., Yarlagadda M., Tzouvelekis A., Gibson K.F., Konishi K., Yousem S.A., Singh M., Handley D. (2010). Inhibition and role of let-7d in idiopathic pulmonary fibrosis. Am. J. Respir. Crit. Care Med..

[133] Wu A., Wu K., Li J., Mo Y., Lin Y., Wang Y., Shen X., Li S., Li L., Yang Z. (2015). Let-7a inhibits migration, invasion and epithelial-mesenchymal transition by targeting HMGA2 in nasopharyngeal carcinoma. J Transl Med.

[134] Sgarra R., Pegoraro S., Ros G., Penzo C., Chiefari E., Foti D., Brunetti A., Manfioletti G. (2018). High Mobility Group A (HMGA) proteins: Molecular instigators of breast cancer onset and progression. Biochim. Biophys. Acta Rev. Cancer.

[135] Huleihel L., Ben-Yehudah A., Milosevic J., Yu G., Pandit K., Sakamoto K., Yousef H., LeJeune M., Coon T.A., Redinger C.J. (2014). Let-7d microRNA affects mesenchymal phenotypic properties of lung fibroblasts. Am. J. Physiol. Lung Cell. Mol. Physiol..

[136] Wang S., Zhou H., Wu D., Ni H., Chen Z., Chen C., Xiang Y., Dai K., Chen X., Li X. (2019). MicroRNA let-7a regulates angiogenesis by targeting TGFBR3 mRNA. J. Cell. Mol. Med..

[137] Yan N., Wen L., Peng R., Li H., Liu H., Peng H., Sun Y., Wu T., Chen L., Duan Q. (2016). Naringenin Ameliorated Kidney Injury through Let-7a/TGFBR1 Signaling in Diabetic Nephropathy. J. Diabetes Res..

[138] Xie H., Gao Y.M., Zhang Y.C., Jia M.W., Peng F., Meng Q.H., Wang Y.C. (2020). Low let-7d exosomes from pulmonary vascular endothelial cells drive lung pericyte fibrosis through the TGFbetaRI/FoxM1/Smad/beta-catenin pathway. J. Cell. Mol. Med..

[139] Skoufos G., Kakoulidis P., Tastsoglou S., Zacharopoulou E., Kotsira V., Miliotis M., Mavromati G., Grigoriadis D., Zioga M., Velli A. (2024). TarBase-v9.0 extends experimentally supported miRNA-gene interactions to cell-types and virally encoded miRNAs. Nucleic Acids Res..

[140] Williams L.M., McCann F.E., Cabrita M.A., Layton T., Cribbs A., Knezevic B., Fang H., Knight J., Zhang M., Fischer R. (2020). Identifying collagen VI as a target of fibrotic diseases regulated by CREBBP/EP300. Proc. Natl. Acad. Sci. USA.

[141] Zhou B., Liu Y., Kahn M., Ann D.K., Han A., Wang H., Nguyen C., Flodby P., Zhong Q., Krishnaveni M.S. (2012). Interactions between beta-catenin and transforming growth factor-beta signaling pathways mediate epithelial-mesenchymal transition and are dependent on the transcriptional co-activator cAMP-response element-binding protein (CREB)-binding protein (CBP). J. Biol. Chem..

[142] Specks U., Nerlich A., Colby T.V., Wiest I., Timpl R. (1995). Increased expression of type VI collagen in lung fibrosis. Am. J. Respir. Crit. Care Med..

[143] Homma S., Nagaoka I., Abe H., Takahashi K., Seyama K., Nukiwa T., Kira S. (1995). Localization of platelet-derived growth factor and insulin-like growth factor I in the fibrotic lung. Am. J. Respir. Crit. Care Med..

[144] Wang L.N., Chen W.W., Zhang J., Li C.Y., Liu C.Y., Xue J., Zhang P.J., Jiang A.L. (2013). The miRNA let-7a1 inhibits the expression of insulin-like growth factor 1 receptor (IGF1R) in prostate cancer PC-3 cells. Asian J. Androl..

[145] Wyss C.B., Duffey N., Peyvandi S., Barras D., Martinez Usatorre A., Coquoz O., Romero P., Delorenzi M., Lorusso G., Ruegg C. (2021). Gain of HIF1 Activity and Loss of miRNA let-7d Promote Breast Cancer Metastasis to the Brain via the PDGF/PDGFR Axis. Cancer Res..

[146] Wang Q., Yu Y., Zhang P., Chen Y., Li C., Chen J., Wang Y., Li Y. (2017). The crucial role of activin A/ALK4 pathway in the pathogenesis of Ang-II-induced atrial fibrosis and vulnerability to atrial fibrillation. Basic Res. Cardiol..

[147] Bo Y., Liu B., Yang L., Zhang L., Yan Y. (2021). Exosomes derived from miR-16-5p-overexpressing keratinocytes attenuates bleomycin-induced skin fibrosis. Biochem. Biophys. Res. Commun..

[148] Jin W., Chen F., Wang K., Song Y., Fei X., Wu B. (2018). miR-15a/miR-16 cluster inhibits invasion of prostate cancer cells by suppressing TGF-beta signaling pathway. Biomed. Pharmacother..

[149] Ma L., Liu J., Xiao E., Ning H., Li K., Shang J., Kang Y. (2021). MiR-15b and miR-16 suppress TGF-beta1-induced proliferation and fibrogenesis by regulating LOXL1 in hepatic stellate cells. Life Sci..

[150] Yao Q., Xing Y., Wang Z., Liang J., Lin Q., Huang M., Chen Y., Lin B., Xu X., Chen W. (2020). MiR-16-5p suppresses myofibroblast activation in systemic sclerosis by inhibiting NOTCH signaling. Aging.

[151] Palmieri D., D’Angelo D., Valentino T., De Martino I., Ferraro A., Wierinckx A., Fedele M., Trouillas J., Fusco A. (2012). Downregulation of HMGA-targeting microRNAs has a critical role in human pituitary tumorigenesis. Oncogene.

[152] Chamorro-Jorganes A., Araldi E., Penalva L.O., Sandhu D., Fernandez-Hernando C., Suarez Y. (2011). MicroRNA-16 and microRNA-424 regulate cell-autonomous angiogenic functions in endothelial cells via targeting vascular endothelial growth factor receptor-2 and fibroblast growth factor receptor-1. Arterioscler. Thromb. Vasc. Biol..

[153] Chen L., Wang Q., Wang G.D., Wang H.S., Huang Y., Liu X.M., Cai X.H. (2013). miR-16 inhibits cell proliferation by targeting IGF1R and the Raf1-MEK1/2-ERK1/2 pathway in osteosarcoma. FEBS Lett..

[154] Zeng N., Wen Y.H., Pan R., Yang J., Yan Y.M., Zhao A.Z., Zhu J.N., Fang X.H., Shan Z.X. (2021). Dickkopf 3: A Novel Target Gene of miR-25-3p in Promoting Fibrosis-Related Gene Expression in Myocardial Fibrosis. J. Cardiovasc. Transl. Res..

[155] Genz B., Coleman M.A., Irvine K.M., Kutasovic J.R., Miranda M., Gratte F.D., Tirnitz-Parker J.E.E., Olynyk J.K., Calvopina D.A., Weis A. (2019). Overexpression of miRNA-25-3p inhibits Notch1 signaling and TGF-beta-induced collagen expression in hepatic stellate cells. Sci. Rep..

[156] Hirota N., McCuaig S., O’Sullivan M.J., Martin J.G. (2014). Serotonin augments smooth muscle differentiation of bone marrow stromal cells. Stem. Cell Res..

[157] Lan Y., Xie H., Jin Q., Zhao X., Shi Y., Zhou Y., Hu Z., Ye Y., Huang X., Sun Y. (2022). Extracellular vesicles derived from neural EGFL-Like 1-modified mesenchymal stem cells improve acellular bone regeneration via the miR-25-5p-SMAD2 signaling axis. Bioact. Mater..

[158] Hu Z., Zhao Y., Jiang J., Li W., Su G., Li L., Ran J. (2023). Exosome-derived miR-142-5p from liver stem cells improves the progression of liver fibrosis by regulating macrophage polarization through CTSB. Environ. Toxicol..

[159] Zhang Y., Xu C. (2021). Integrative analysis of miRNA-mRNA expression profiles in esophageal fibrosis after ESD. Exp. Ther. Med..

[160] Narasimhan M., Patel D., Vedpathak D., Rathinam M., Henderson G., Mahimainathan L. (2012). Identification of novel microRNAs in post-transcriptional control of Nrf2 expression and redox homeostasis in neuronal, SH-SY5Y cells. PLoS ONE.

[161] Liang W., Yang H., Pan L., Wei S., Li Z., Zhang P., Li R., Wu Y., Liu M., Liu X. (2023). Ginkgo biloba Extract 50 (GBE50) Exerts Antifibrotic and Antioxidant Effects on Pulmonary Fibrosis in Mice by Regulating Nrf2 and TGF-beta1/Smad Pathways. Appl. Biochem. Biotechnol..

[162] Hua Q., Ren L. (2024). The SIRT1/Nrf2 signaling pathway mediates the anti-pulmonary fibrosis effect of liquiritigenin. Chin. Med..

[163] Huang C., Xiao X., Yang Y., Mishra A., Liang Y., Zeng X., Yang X., Xu D., Blackburn M.R., Henke C.A. (2017). MicroRNA-101 attenuates pulmonary fibrosis by inhibiting fibroblast proliferation and activation. J. Biol. Chem..

[164] Vuga L.J., Ben-Yehudah A., Kovkarova-Naumovski E., Oriss T., Gibson K.F., Feghali-Bostwick C., Kaminski N. (2009). WNT5A is a regulator of fibroblast proliferation and resistance to apoptosis. Am. J. Respir. Cell Mol. Biol..

[165] Newman D.R., Sills W.S., Hanrahan K., Ziegler A., Tidd K.M., Cook E., Sannes P.L. (2016). Expression of WNT5A in Idiopathic Pulmonary Fibrosis and Its Control by TGF-beta and WNT7B in Human Lung Fibroblasts. J. Histochem. Cytochem..

[166] Yang J., Lu Y., Lin Y.Y., Zheng Z.Y., Fang J.H., He S., Zhuang S.M. (2016). Vascular mimicry formation is promoted by paracrine TGF-beta and SDF1 of cancer-associated fibroblasts and inhibited by miR-101 in hepatocellular carcinoma. Cancer Lett..

[167] Pulito-Cueto V., Genre F., Lopez-Mejias R., Mora-Cuesta V.M., Iturbe-Fernandez D., Portilla V., Sebastian Mora-Gil M., Ocejo-Vinyals J.G., Gualillo O., Blanco R. (2023). Endothelin-1 as a Biomarker of Idiopathic Pulmonary Fibrosis and Interstitial Lung Disease Associated with Autoimmune Diseases. Int. J. Mol. Sci..

[168] Shahar I., Fireman E., Topilsky M., Grief J., Schwarz Y., Kivity S., Ben-Efraim S., Spirer Z. (1999). Effect of endothelin-1 on alpha-smooth muscle actin expression and on alveolar fibroblasts proliferation in interstitial lung diseases. Int. J. Immunopharmacol..

[169] Rizvi M.A., Katwa L., Spadone D.P., Myers P.R. (1996). The effects of endothelin-1 on collagen type I and type III synthesis in cultured porcine coronary artery vascular smooth muscle cells. J. Mol. Cell. Cardiol..

[170] Marini M., Carpi S., Bellini A., Patalano F., Mattoli S. (1996). Endothelin-1 induces increased fibronectin expression in human bronchial epithelial cells. Biochem. Biophys. Res. Commun..

[171] Zhu W., Liu C., Tan C., Zhang J. (2024). Predictive biomarkers of disease progression in idiopathic pulmonary fibrosis. Heliyon.

[172] Yan L., Su Y., Hsia I., Xu Y., Vincent-Chong V.K., Mojica W., Seshadri M., Zhao R., Wu Y. (2023). Delivery of anti-microRNA-21 by lung-targeted liposomes for pulmonary fibrosis treatment. Mol. Ther. Nucleic Acids.

[173] Liang H., Liu S., Chen Y., Bai X., Liu L., Dong Y., Hu M., Su X., Chen Y., Huangfu L. (2016). miR-26a suppresses EMT by disrupting the Lin28B/let-7d axis: Potential cross-talks among miRNAs in IPF. J. Mol. Med..

[174] Raghu G., Remy-Jardin M., Ryerson C.J., Myers J.L., Kreuter M., Vasakova M., Bargagli E., Chung J.H., Collins B.F., Bendstrup E. (2020). Diagnosis of Hypersensitivity Pneumonitis in Adults. An Official ATS/JRS/ALAT Clinical Practice Guideline. Am. J. Respir. Crit. Care Med..

[175] Arroyo J.D., Chevillet J.R., Kroh E.M., Ruf I.K., Pritchard C.C., Gibson D.F., Mitchell P.S., Bennett C.F., Pogosova-Agadjanyan E.L., Stirewalt D.L. (2011). Argonaute2 complexes carry a population of circulating microRNAs independent of vesicles in human plasma. Proc. Natl. Acad. Sci. USA.

[176] Moldovan L., Batte K., Wang Y., Wisler J., Piper M. (2013). Analyzing the circulating microRNAs in exosomes/extracellular vesicles from serum or plasma by qRT-PCR. Methods Mol. Biol..

[177] Espina-Ordonez M., Balderas-Martinez Y.I., Torres-Machorro A.L., Herrera I., Maldonado M., Romero Y., Toscano-Marquez F., Pardo A., Selman M., Cisneros J. (2024). Mir-155-5p targets TP53INP1 to promote proliferative phenotype in hypersensitivity pneumonitis lung fibroblasts. Noncoding RNA Res..

[178] Tomos I., Roussis I., Matthaiou A.M., Dimakou K. (2023). Molecular and Genetic Biomarkers in Idiopathic Pulmonary Fibrosis: Where Are We Now?. Biomedicines.

[179] Stainer A., Faverio P., Busnelli S., Catalano M., Della Zoppa M., Marruchella A., Pesci A., Luppi F. (2021). Molecular Biomarkers in Idiopathic Pulmonary Fibrosis: State of the Art and Future Directions. Int. J. Mol. Sci..

[180] Vlachos I.S., Zagganas K., Paraskevopoulou M.D., Georgakilas G., Karagkouni D., Vergoulis T., Dalamagas T., Hatzigeorgiou A.G. (2015). DIANA-miRPath v3.0: Deciphering microRNA function with experimental support. Nucleic Acids Res..

[181] Quillet A., Saad C., Ferry G., Anouar Y., Vergne N., Lecroq T., Dubessy C. (2019). Improving Bioinformatics Prediction of microRNA Targets by Ranks Aggregation. Front. Genet..

[182] Zhou Z., Xie Y., Wei Q., Zhang X., Xu Z. (2024). Revisiting the role of MicroRNAs in the pathogenesis of idiopathic pulmonary fibrosis. Front. Cell Dev. Biol..

[183] Huang C., Yang Y., Liu L. (2015). Interaction of long noncoding RNAs and microRNAs in the pathogenesis of idiopathic pulmonary fibrosis. Physiol. Genomics.

[184] Shevchenko G., Morris K.V. (2018). All I’s on the RADAR: Role of ADAR in gene regulation. FEBS Lett..

